# Infection Efficiency of Four *Phytophthora infestans* Clonal Lineages and DNA-Based Quantification of Sporangia

**DOI:** 10.1371/journal.pone.0136312

**Published:** 2015-08-24

**Authors:** Mamadou Lamine Fall, David Mathieu Tremblay, Mélanie Gobeil-Richard, Julie Couillard, Hélène Rocheleau, Hervé Van der Heyden, Camile André Lévesque, Carole Beaulieu, Odile Carisse

**Affiliations:** 1 Department of Biology, University of Sherbrooke, Sherbrooke, Quebec, Canada; 2 Horticulture Research and Development Centre, Agriculture and Agri-Food Canada, St-Jean-sur-le-Richelieu, Quebec, Canada; 3 Compagnie de recherche Phytodata inc., Sherrington, Quebec, Canada; 4 Central Experimental Farm, Agriculture and Agri-Food Canada, Ottawa, Ontario, Canada; University of Nebraska-Lincoln, UNITED STATES

## Abstract

The presence and abundance of pathogen inoculum is with host resistance and environmental conditions a key factor in epidemic development. Therefore, several spore-sampling devices have been proposed to monitor pathogen inoculum above fields. However, to make spore sampling more reliable as a management tool and to facilitate its adoption, information on infection efficiency and molecular tools for estimating airborne sporangia concentration are needed. Experiments were thus undertaken in a growth chamber to study the infection efficiency of four clonal lineages of *P*. *infestans* (US-8, US-11, US-23, and US-24) by measuring the airborne sporangia concentration and resulting disease intensity. The relationship between the airborne sporangia concentration and the number of lesions per leaf was exponential. For the same concentration, the sporangia of US-23 caused significantly more lesions than the sporangia of the other clonal lineages did. Under optimal conditions, an airborne sporangia concentration of 10 sporangia m^−3^ for US-23 was sufficient to cause one lesion per leaf, whereas for the other clonal lineages, it took 15 to 25 sporangia m^−3^ to reach the same disease intensity. However, in terms of diseased leaf area, there was no difference between clonal lineages US-8, US-23 and US-24. Also, a sensitive quantitative real-time polymerase chain reaction (qPCR) tool was developed to quantify *P*. *infestans* airborne sporangia with detection sensitivity of one sporangium. The specificity of the qPCR assay was rigorously tested for airborne inoculum and was either similar to, or an improvement on, other published PCR assays. This assay allows rapid and reliable detection and quantification of *P*. *infestans* airborne sporangia and thereby, facilitates the implementation of spores-sampling network.

## Introduction

Potato late blight epidemics have become increasingly problematic in many parts of the world [1–3]. *Phytophthora infestans*, the causal agent of late blight disease in solanaceous plants, has been under investigation for the past 150 years. The disease spreads very quickly under cool, humid conditions when sporangia are produced on infected leaves and further splashed or wind-blown onto nearby plants [2]. Worldwide losses caused by late blight have been estimated at billions of U.S. dollars each year [3]. Management strategies for late blight are based essentially on the timing and efficiency of fungicide applications. Thus, most fungicide applications are timed using decision-support systems (DSSs), which target the optimal moment for fungicide application [4, 5].

Several DSSs that forecast potato late blight have been developed to guide decision making about fungicide applications in different potato production areas around the world [6]. These systems, which are used for predicting the risk of infection, are considered useful tools for controlling late blight with fewer applications of chemical fungicides than fixed-interval fungicide spray programs [6,7]. However, the major constraint of DSSs is the absence of information on the presence and the quantity of airborne inoculum in potato fields. Consequently, DSSs can overestimate or underestimate late blight risk [8]. Recent studies suggested that including the information from monitoring of *P*. *infestans* airborne inoculum could improve DSSs [5, 8]. Those studies suggested that combining a spore-sampling network with a DSS could potentially aid targeting the optimal time to apply a disease-control product [5, 8].

The potential consequences of late blight infection force growers to frequently spray their crops from the time the plants meet between rows all the way until harvest. This situation has been addressed by combining management strategies such as using resistant cultivars, altering planting practices, using certified seed, using DSSs, measuring airborne inoculum etc. [5, 8, 9]. However, to rationalize fungicide use and minimize the build-up of fungicide resistance, these measures should be applied in a coordinated and integrated manner. For example, airborne sporangia can be a counterweight to the DSS risk estimate: a high risk combined with a significant airborne sporangia concentration (ASC) will trigger fungicide spraying [8]. Therefore, the link between ASC and late blight intensity, along with the variation in disease intensity between different clonal lineages of *P*. *infestans*, should be studied. In North America, the sexual reproduction of *P*. *infestans* is limited, and thus the cycle of aerial asexual sporangia dispersal plays a crucial role in late blight development [10, 11]. Also, increases in the intensity of potato late blight in North America coincide with major genetic changes in the *P*. *infestans* population [12]: in 2009, for example, many producers lost practically all of their crops mostly because of the rapid spread of new clonal lineage of *P*. *infestans* and fungicide resistance [1]. Because they seem to present different epidemiological characteristics [10, 12], clonal lineages are expected to be distinctive in terms of infection efficiency. In this context, infection efficiency is defined as the ratio of late blight intensity (number of lesions per leaf or disease severity) to ASC. Moreover, to improve the reliability of the information derived from measurements of airborne inoculum, a reliable quantitative tool is needed to accurately estimate inoculum concentrations.

The conventional method for estimation of *P*. *infestans* airborne sporangia is based on counting under microscope, which is time consuming, especially in the context of field sampling for rapid decision making. Instead methods for molecular quantification of *P*. *infestans* airborne inoculum could be developed. The sequence of the *P*. *infestans* genome is now complete and available [13], and thus numerous molecular tools have been developed [14–15] for use not only to understand the mechanisms that regulate the organism’s survival and resistance responses or pathogenesis but also to conduct genotyping and phenotyping studies [16]. Over the past 20 years, several molecular tools for detecting or quantifying *Phytophthora* propagules on leaves and soil have been developed and used to improve late blight management [15–17, 18–19].

The disease triangle is a fundamental and unique principle describing factors involved in plant disease causation. Indeed, plant disease is prevented upon elimination of any one of the three components of disease triangle, which are, a susceptible host, a pathogen, and a favourable environment (e.g. weather conditions) [20]. Considerable efforts have been made to identify or develop late blight-resistant varieties and reliable weather forecasting models. However, in integrated manner presence and abundance of pathogen inoculum is at least as important as host resistant and environmental conditions. Over the past 25 years, several spore-sampling devices have been proposed to monitor the pathogen inoculum above fields. Therefore, spore-sampling networks have been successfully implemented at a regional scale in different production area around the world [5–8,21,22]. For example, farmers in different potato production area in Canada are using information derived from spore-sampling network as functional strategy to control late blight epidemics [8]. Similar network were successfully implemented in organic soil in southwest of Montreal to manage leaf blight of onion and lettuce downy mildew [8, 20]. Also, In Michigan, field advisers monitor airborne *Venturia inaequalis* ascospores in apple orchards with spore samplers and use automated information lines, fax networks, and e-mail to keep producers informed [8]. Nevertheless, in order to facilitate interpretation of information derived from spore-sampling for decision-making, further study should be done to determine the link between the airborne spore concentration and disease intensity (action threshold), to develop a molecular tool for quantification of airborne spore, and to determine the number of samplers required depending on the size of the production area. This situation led to the hypothesis that there is a link between ASC and late blight intensity. Hence, the specifics objectives of this study were (i) to determine the infection efficiency of four clonal lineages of *P*. *infestans* and compare late blight intensity among them and (ii) to develop a TaqMan qPCR assay for the detection and quantification of airborne sporangia of *P*. *infestans*. This study shows an exponential relationship between *P*. *infestans* airborne sporangia concentration and the number of lesions per potato leaf. As expected, the infection efficiency varies among *P*. *infestans* clonal lineages. Also, a sensitive quantitative polymerase chain reaction (qPCR) assay that can detect low concentrations of *P*. *infestans* sporangia in air samples was developed. These results are expected to improve and facilitate interpretation of information derived from the spore-sampling network.

## Materials and Methods

Agriculture and AgriFood Canada (AAFC) provided the experimental infrastructure for each activity in this study and no specific permissions were required for these activities. Also, the study did not involve endangered or protected species. All data are available in Tables, Figures and Supporting Information files.

### Data collection to determine the infection efficiency of *P*. *infestans* clonal lineages

#### Experimental design

Unconventional assay was designed in a growth chamber to investigate the relationship between airborne inoculum and late blight intensity. A proof of concept of this essay was done in a previous study [22]. A total of 20 potato plants (cv. Russet Burbank) that had been produced in a greenhouse and had reached the 10-to-13-leaf stage were placed 0.15 m apart on the bottom of a growth chamber (PGC20 growth chamber; Conviron, Winnipeg, MB, Canada). In the upper part of the chamber, 4-to-8 potato leaves that had sporulating lesions on them and that had been produced from inoculated plants maintained in the growth chamber were placed 1.20 m above the potato plants so that the plants would be infected by airborne sporangia in a manner similar to infection under field conditions. To promote infection, the conditions in the growth chamber were 15 h of darkness, 9 h of daylight, 18°C, 100% relative humidity, and a leaf-wetness period of 6 h. While the sporulating lesions were in the growth chamber, a rotating-arm spore sampler (Compagnie de recherche Phytodata inc., Sherrington, QC, Canada) placed 0.5 m above the potato plant canopy was used to monitor sporangia concentrations. The sampler ran 30% of time during the 15 h of darkness for a total of 4 h (alternating between 4 min on and 11 min off), and the effective air-sampling rate was 21.65 L/min. After 14 h, the sporulating leaves were removed, and the growth chamber was maintained at 18°C and 90% relative humidity for 6 d in a cycle of 9 h of darkness and 15 h of daylight. The ASCs were also monitored to make sure that no airborne sporangia remained in the growth chamber one hour after the sporulating leaves had been removed. Sporangia caught on the sampling surfaces (rods) were counted within 24 h after sampling with a microscope at 250× magnification. The number of sporangia per rod was converted to sporangia per cubic meter of air as follows: (number of sporangia per rod × 1,000 L/m^3^/h)/(21.65 L/min per rod × 60 min/h × 4 h). This experiment was repeated 14, 16, 14, and 17 times for clonal lineages US-24, US-23, US-11, and US-8, respectively. To obtain a range of airborne sporangia concentration, the number of sporulating lesions that were introduced in the growth chamber was different in each assay. Airborne sporangia concentrations ranging between 0 (no sporulating lesions) and 40 sporangia m^−3^ were measured for each of the clonal lineages. The control for each lineage was the first experimental run without sporulating lesions in the growth chamber (control). To evaluate the uniformity of sporangial distribution in the growth chamber, sporangia collected on microscope slides placed in the bottom of the growth chambers were counted and compared.

All clonal lineages were collected in Canada, the clonal lineages US-23, US-24 and US-8 were kindly provided by Dr. Khalil Al-Mughrabi (New Brunswick Department of Agriculture, Aquaculture and Fisheries) and US-11 was provided by Dr. Katherine Dobinson (Agriculture and Agri-Food Canada, southern crop protection and food research center). For each clonal lineage, a mixture of three isolates was used to infect potato plants and produce sporulating lesions.

#### Measurement of disease

Seven day after each inoculation, the number of lesions per leaf and disease severity were assessed on all trap plants by randomly selecting five leaves on each plant. Disease severity was recorded by visual estimation of the leaf area with late blight lesions. Severity was estimated on a scale from 1 to 10, where: 1 = no lesion; 2 = 0^+^__ 3%; 3 = 3^+^__ 5%; 4 = 5^+^__10%; 5 = 10^+^__25%; 6 = 25^+^__50%; 7 = 50^+^__75%; 8 = 75^+^__85%; 9 = 85^+^__95% and 10 = 95^+^__100% of leaf area with late blight symptoms [23, 24]. The superscript (+) means value just slightly above the indicated value.

#### Data analysis

Before analysis, the disease severity rating was converted to the severity percentage using the interval midpoint [25], as follows: 1 = 0%, 2 = 1.5%, 3 = 4%, 4 = 7.5%, 5 = 17.5%, 6 = 37.5%, 7 = 62.5%, 8 = 80%, 9 = 90%, and 10 = 97.5%. The ASCs were grouped into the following three categories: low for ASC ≤ 5 sporangia m^−3^, medium for 5 < ASC ≤ 10 sporangia m^−3^, and high for ASC > 10 sporangia m^−3^. First, normality of the data was tested using the Shapiro–Wilk test, and then, if necessary, logarithmic transformation was done to improve normality. All statistical tests were performed in R software (version 3.0.0; R Foundation for Statistical Computing, Vienna, Austria).

#### Infection efficiency of *P*. *infestans* clonal lineages

A scatter plot representing the number of lesions per leaf as a function of ASC was prepared. To describe the relationship between the number of lesions per leaf and ASC, a two-parameter exponential rise to a maximum function was fitted to the data, as follows:

y=a(1−exp(−bx)) (1)

where *y* is the number of lesions per leaf, *x* is the ASC, *a* is the asymptote, and *b* is the rate. The model’s appropriateness and goodness of fit to the data were assessed using an adjusted *R* ^2^ [25].

A scatter plot representing the severity (percentage of leaf area diseased) as a function of ASC was prepared. To describe the relationship between those two variables, a linear function with an ordinate intercept of zero was fitted to the data. Two factors were considered, ASC and clonal lineage. ASC is a factor with three levels: low, medium and high. Clonal lineage is a factor with four levels: US-8, US-11, US-23 and US-24. A factorial analysis of variance (ANOVA) was used to investigate statistical interactions, in which the number of lesions caused by clonal lineages depends on the level of ASC. Because the sample size for each level differed (unbalanced design), the factors were entered in different orders to see how much of a difference this makes to the outcomes of the analysis. The agricolae package in R (agricolae: Statistical Procedures for Agricultural Research, version 1.1–4, in statistical software R3.0.0 GUI 1.60; Lima, Peru) was used [26]. When the full model was statistically significant (α = 0.05), means across the number of lesions per leaf among clonal lineages were compared, using the Tukey HSD (honest significant difference) post hoc comparison. A pairwise *t*-test was used to calculate the adjusted *P*-values (agricolae: Statistical Procedures for Agricultural Research) [26].

### Development of the *P*. *infestans* real-time qPCR assay

#### Design of primers

Multiple sequence alignments were built from sequences originating from internal transcribed spacer (ITS) DNA segments of different oomycetes [27]. A short region with high dissimilarities among the *Phytophthora* species was identified within the ITS2 region. This DNA segment was submitted to the PrimerQuest tool (http://www.idtdna.com/Primerquest/Home/Index). The newly designed primers and the TaqMan probe set ITS2HR-pinf-F, ITS2HR-pinf-R, and ITS2HR-pinf-P amplified a 108-base-pair (bp) product and are listed in Table 1.

**Table 1:** Primer and probe names and sequences for qPCR reactions.

Primer/probe/sequence name	5′-3′ sequence
**ITS2HR-pinf-F**	TGG ACT GGT GAA CCA TGG CTC TT
**ITS2HR-pinf-P^a^**	TTG CGA AGT AGA GTG GCG GCT TCG GCT GC
**ITSHR-pinf-R**	CAA CAT TTC CCA AAT GGA TCG ACC CT
**PITSstdF**	AACTAGATAGCAACTTTC
**PITSstdR**	GTTTTCAGGTACTCTTTA
**EIPC100-F**	AGGCTAGCTAGGACCGATCAATAGG
**EIPC100-P^b^**	CCTATGCGTTCCGAGGTGACGACCTTGCC
**EIPC100-R**	AGTGCTTCGTTACGAAAGTGACCTTA

^a^ Zen double-quenched fluorescent-labelled probe 5′-6-Fam, 3′-IBHQ1.

^b^ Zen double-quenched fluorescent-labelled probe 5′-HEX, 3′-IBHQ1.

#### Exogenous internal positive control

To detect partial or complete inhibition of the qPCR reaction, as well as potential experimental errors, an exogenous internal positive control (EIPC) was developed and added in the DNA extraction and dilution solutions. The EIPC fragment (S1 Table) consisted of double-stranded DNA genomic blocks (gBlocks; Integrated DNA Technologies, Inc., Coralville, IA, USA) designed from a random DNA sequence of 500 bp generated from Stothard P (2000) (http://www.bioinformatics.org/sms2/reference.html). Primers EIPC100-F and EIPC100-R and TaqMan probe EIPC100-P (Table 1) were designed from the EIPC sequence using Beacon Designer 8.13 (Premier Biosoft, Palo Alto, CA, USA) and generated a 100-bp amplicon.

#### Preparation of DNA solution for construction of *P*. *infestans* ITS2 copy standard curve

DNA from *P*. *infestans* isolates (US-6, US-8, US-11, US-22, US-23, and US-24) was used for the PCR amplification of a 1004-bp ITS2 fragment. In a 50-μL reaction mixture containing 6 ng of genomic DNA, 1X Taq PCR buffer, 4.0 mM of MgCl^2+^, 0.03 U/μL of SurePRIME Taq polymerase (MP Biomedicals, Solon, OH, USA), 0.4 mM of dNTPs (New England Biolabs Inc., Ipswich, MA, USA), and 200 nM each of primers PITSstdF and PITSstdR (Table 1), the following PCR conditions were used: 95°C for 15 min, followed by 40 cycles at 94°C for 15 s, 58°C for 45 s, and 72°C for 1 min, and a final extension at 72°C for 10 min. The PCR fragments were purified using the Nucleospin PCR clean-up kit (Macherey-Nagel GmbH & Co., Düren, Germany) according to the manufacturer’s recommendations. The purified products were quantified using a 2100 Bioanalyzer instrument (Agilent Technologies, Santa Clara, CA, USA). The DNA was analyzed according to the manufacturer’s instructions and converted into copy numbers, calculated as copy number = [(concentration of amplicon in g/μL)/(1004 bp × 660 g/mole) × (6.022 × 10^23^)]. This DNA was then used for optimizing the TaqMan qPCR assay and generating the ITS2 copy number standard curve.

#### Preparation of DNA solution for construction of *P*. *infestans* sporangia standard curve

To prepare the sporangial standard curve, dry sporangia of US-8 and US-11 were harvested from sporulating potato leaves. The sporangia were harvested from four to six fresh sporulating lesions, avoiding the necrotic area, using a truncated disposable 10-mL pipette connected to a vacuum pump. Approximately 5 mL of isopropanol 100% (Sigma-Aldrich Canada Ltd.) was then poured into the pipette to suspend the harvested sporangia. Suspensions of 3125 sporangia were transferred into 2-mL screw-cap tubes containing 100 mg of 425-to-600-μm glass beads (Sigma-Aldrich Canada Ltd, Oakville, ON, Canada). The isopropanol was then evaporated using a Vacufuge (Eppendorf Canada Ltd., Mississauga, Ontario, Canada) at 60°C for 20 min and then the tubes were stored at −20°C until DNA extraction. To maximize the quantity of DNA pulled out, a lossless DNA extraction method without purification was used. Lysis of sporangia and DNA recovery were accomplished in a single extraction tube containing a silicon-coated spore trap rod and 75 μl of ACS reagent-grade petroleum ether (Sigma-Aldrich). A silicon coated polystyrene spore trap rod was added to the tubes to mimic same lysis conditions that field collected samples as discussed later in this manuscript. The tubes were shaken for 20 s at 4 m/s using a Fast-Prep instrument (MP Biomedicals, Solon, Ohio) and then centrifuged for 5 s at 10,000 x g to pellet the sporangial debris at the bottom of the tubes. Petroleum ether was evaporated using the Vacufuge instrument for 20 min at 60°C. The sporangial lysate was suspended in 300 μL of DNA extraction solution consisting nuclease-free water, salmon sperm DNA at 10 ng/μL (Life Technologies Inc., Burlington, ON, Canada), 5% Chelex 100 (Bio-Rad Laboratories Ltd., Mississauga, ON, Canada), 100 ng/μL bovine serum albumin (New England Biolabs Inc.), and an EIPC fragment at 2 × 10^2^ copies/μL. To complete DNA extraction, the tubes were then heated at 105°C for 20 min in a dry bath, briefly agitated using a vortex, and centrifuged at 4°C and 15,000 × *g*. The supernatants were kept at 4°C for a maximum of 1 h to avoid DNA degradation. The DNA was then used for optimizing the TaqMan qPCR assay and constructing the *P*. *infestans* sporangia standard curve.

#### Construction of standard curves to estimate the number of spores from the number of ITS2 copies

The ITS2 copy standard curve was constructed by 10-fold serial dilutions of the 1004 bp *P*. *infestans* ITS-2 gene fragment, ranging from 1 × 10^5^ to 1 copy/μL in a DNA dilution solution consisting of nuclease-free water containing salmon sperm DNA at 10 ng/μL and EIPC at 2 × 10^2^ copies/μL. The sporangia standard curve was constructed by five-fold serial dilutions of 3125 to one *P*. *infestans* sporangium in the DNA dilution solution as described above. For both curves, the procedure was repeated three times. A three-step regression analysis was used to determine the number of copies of ITS2 per sporangia. First, regression analysis was used to establish the relationship between the quantification cycle threshold (Cq) value and the logarithm of the number of ITS2 copies. Second, regression analysis was used to establish the relationship between the Cq value and the logarithm of the number of sporangia. Finally, the predicted number of sporangia was regressed against the predicted number of DNA fragments for Cq values of 18 to 30. The resulting equation was used to determine the number of sporangia from the number of copies obtained from the qPCR assay. The regression procedure (PROC REG) of SAS (version 9.1; SAS Institute Inc., Cary, NC, USA) was used to estimate the intercept and slope regression parameters.

#### Extraction procedure of *P*. *infestans* DNA from field sampler

*Phytophthora*. *infestans* DNA from field collected spore trap polystyrene rods were extracted using lossless DNA extraction as described previously. However, during the first step of the procedure, single polystyrene rods were placed directly in each extraction tube with 75 μl of petroleum ether instead of suspending sporangia in isopropanol. Supernatants were then maintained at 4°C for a maximum of 1 h and used as the DNA solution for qPCR sporangia assessment.

#### Evaluation of the TaqMan qPCR assay procedure

A standard curve of the *P*. *infestans* 1004-bp *P*. *infestans* fragment was established as described above in the DNA dilution solution. The reactions were performed in a 25 μL final volume containing 5 μL of DNA extract, 12.5 μL of 2x QuantiFast Multiplex Probe PCR Master Mix (Qiagen Inc, Mississauga, ON, Canada), 300 nM each of primers ITS2HR-pinf-F, ITS2HR-pinf-R, EIPC100-F and EIPC100-R and 200 nM each of TaqMan probes ITS2HR-pinf-P and EIPC100-P. The two-step PCR conditions were 95°C for 5 min, followed by 40 cycles at 95°C for 30 s and 62°C for 30 s in a Mx3005P qPCR Instrument (Agilent Technologies, Santa Clara, CA, USA). The results were evaluated for the efficiency of *P*. *infestans* amplification and the stability of EIPC detection among the dilutions.

#### Specificity of the qPCR assay

The specificity of the TaqMan qPCR assay was tested on several species of *Phytophthora* as well as on *Pythium vexans* (Table 2). The TaqMan qPCR assay was performed as described previously using 100 pg of genomic DNA added to the 25-μL assay reaction. The qPCR reaction was performed using the QuantiFast Multiplex PCR +R Kit (Qiagen Inc.). The two-step qPCR conditions were the same as those described above.

**Table 2:** Isolates/DNA of species used in this investigation and evaluation of species specificity of the TaqMan assay. Fluorescence is indicated as detected (+) or undetected (−) for *Phytophthora* species.

Isolate/DNA	Species	Host	Origin	PCR reaction	Provided by
**Pi 09-30-DO1**	*P*. *infestans* (US-6)	*Solanum tuberosum*	Prince Edward Island, Canada	+	1
**Pi 09-09-A03**	*P*. *infestans* (US-8)	*Solanum tuberosum*	Prince Edward Island, Canada	+	1
**Pi 281-P3C10**	*P*. *infestans* (US-8)	*Solanum tuberosum*	Prince Edward Island, Canada	+	1
**Pi 31-CO1**	*P*. *infestans* (US-11)	*Solanum tuberosum*	Prince Edward Island, Canada	+	1
**Pi-Rusinek**	*P*. *infestans* (US-22)	*Solanum tuberosum*	Ithaca, New York, USA	+	2
**Pi-09-225-P3B10**	*P*. *infestans* (US-8)	*Solanum tuberosum*	Ithaca, New York, USA		2
**Pi-US23NB**	*P*. *infestans* (US-23)	*Solanum tuberosum*	New Brunswick, Canada	+	3
**Pi-US24NB**	*P*. *infestans* (US-24)	*Solanum tuberosum*	New Brunswick, Canada	+	3
**Pi-3-1-11**	*P*. *infestans* (US-24)	*Solanum tuberosum*	Manitoba, Canada	+	4
**Pi-4-1-11**	*P*. *infestans* (US-24)	*Solanum tuberosum*	Manitoba, Canada	+	4
**P12 (P1B10)**	*P*. *capsici*	N/A	Ithaca, New York, USA	−	2
**H7 (P2B10)**	*P*. *capsici*	N/A	Ithaca, New York, USA	−	2
**P13365**	*P*. *andina*	*Solanum brevifolium*	Ecuador	+	5
**P3008**	*P*. *mirabilis*	*Mirabilis jalapa*	Mexico	+	5
**P6609**	*P*. *phaseoli*	*Phaseolus lunatus*	Maryland, USA	+	5
**N/A**	*P*. *ipomoeae*	*Ipomoea longipedunculata*	Maryland, USA	+	5
**P10566**	*P*. *alni*	*Alnus glutinosa*	Hungary	−	5
**N/A**	*P*. *kernoviae*	N/A	Maryland, USA	−	5
**N/A**	*P*. *alni*	*Alnus* spp.	Maryland, USA	−	5
**P10207**	*P*. *cactorum*	N/A	N/A	−	5
**N/A**	*P*. *megasperma*	*Glycine* spp.	N/A	−	5
**P1699**	*P*. *erythroseptica*	*Solanum tuberosum*	Ohio, USA	−	5
**P1435**	*P*. *fragariae*	*Fragaria* × *ananassa*	England	−	5
**N/A**	*P*. *pseudotsugae*	N/A	Maryland, USA	−	5
**P0991**	*P*. *nicotianae*	*Citrus* spp.	California, USA	−	5
**N/A**	*P*. *ideai*	Rubus occidentalis	California, USA	−	5
**P10303**	*P*. *ramorum*	*Rhododendron* spp.	Netherlands	−	5
**P6497**	*P*. *sojae*	*Glycine max*	Mississippi, USA	−	5
**P0767**	*P*. *citricola*	*Syringa* spp.	Canada	−	5
**N/A**	*P*. *clandestina*	N/A	Maryland, USA	−	5
**P3281**	*P*. *inundata*	*Rubus idaeus*	New York, USA	N/A	5
**P7889**	*P*. *medicagae*	*Trifolium repens* L.	New York, USA	N/A	5
**N/A**	*P*. *iranica*	Myrtus nivellei	Maryland, USA	−	5
**P2021**	*P*. *cinnamomi*	*Camellia japonica*	California, USA	−	5
**N/A**	*P*. *tentaculata*	*Mimulus aurantiacus*	Maryland, USA	−	5
**P1703**	*P*. *crypthogea*	*Solanum tuberosum*	Ohio, USA	N/A	5
**P3273**	*P*. *brassicae*	*Brassica oleracea*	Netherlands	−	5
**P10190**	*P*. *meadii*	*Citrus* spp.	India	N/A	5
**N/A**	*P*. *inflate*	*Ulmus* spp.	Maryland, USA	−	5
**N/A**	*P*. *psychrophila*	N/A	Maryland, USA	−	5
**P15550**	*P*. *quercina*	*Quercus rubra*	Minnesota, USA	−	5
**P10695**	*P*. *insolita*	*Asparagus officinalis*	California, USA	N/A	5
	*P*. *palmivora*	*Cocos nucifera*	Maryland, USA	N/A	5
**P15553**	*P*. *europaea*	*Quercus alba*	West Virginia, USA	−	5
**N/A**	*P*. *arecae*	*Citrus* spp.	Maryland, USA	−	5
**P0592**	*P*. *cambivora*	*Abies procera*	Oregon, USA	N/A	5
**P3849**	*P*. *drechsleri*	*Actinidia deliciosa*	California, USA	N/A	5
**A1000**	*P*. *heveae*	*Rhododendron* spp.	North Carolina, USA	N/A	5
**P3939**	*P*. *ilicis*	*Ilex* spp.	British Columbia, Canada	N/A	5
**N/A**	*Pythium vexans*	*Citrus* spp.	Maryland, USA	−	5
**P1462**	*P*. *trifolii*	*Trifolium vesiculosum*	Mississippi, USA	N/A	5
**P7293**	*P*. *citrophthora*	*Rhododendron* sp	Massachusetts, USA	−	5
**P11061**	*P*. *hedraiandra*	*Viburnum tinus*	Balearic Islands, Spain	−	5
**P6137**	*P*. *gonapododyides*	*Pseudotsuga menziesii*	Oregon, USA	N/A	5
**P6404**	*P*. *rubi*	*Rubus idaeus*	West Germany	N/A	5
**P0647**	*P*. *hibernalis*	*Citrus sinensis*	California, USA	N/A	5
**P10971**	*P*. *foliorum*	*Rhododendron* spp.	California, USA	N/A	5
**P16355**	*P*. *pseudosyringae*	*Umbellularia californica*	California, USA	−	5
**P1372**	*P*. *katsurae*	*Cocos nucifera*	Hawaii, USA	−	5
***P*. *lateralis***	*P*. *lateralis*	N/A	N/A	N/A	6
***P*. *syringae***	*P*. *syringae*	N/A	N/A	−	6

1 Dr. Bud Platt Agriculture and Agri-Food, Prince Edward Island, Canada

2 Dr. Bill Fry, Cornell University, Ithaca, NYC, USA

4 Dr. Fouad Daayf, University of Manitoba, Winnipeg, Manitoba

5 Dr. Michael Coffey, University of California Riverside, Riverside, California, USA

6 Dr. Schmale, Virginia Polytechnic Institute and State University, Blacksburg, Virginia, USA

### Laboratory validation of the qPCR assay

Drops of a *P*. *infestans* sporangia solution in isopropanol 100% (as described above) were deposited onto glass rods coated with silicon vacuum grease [21]. The rods were kept at room temperature for about 5 min to allow the isopropanol to evaporate. The number of spores per rod was counted under a light microscope at 250× magnification. DNA was extracted as described above, and the number of spores was estimated with the qPCR assay and standard curve as described above. The experiment was repeated 23 times. Regression analysis was used to establish the relationship between the number of sporangia estimated by qPCR and the number estimated by microscopy. The analysis was performed using the regression procedure.

## Results

### Relationship between airborne sporangia concentration and late blight intensity

**Fig 1:**
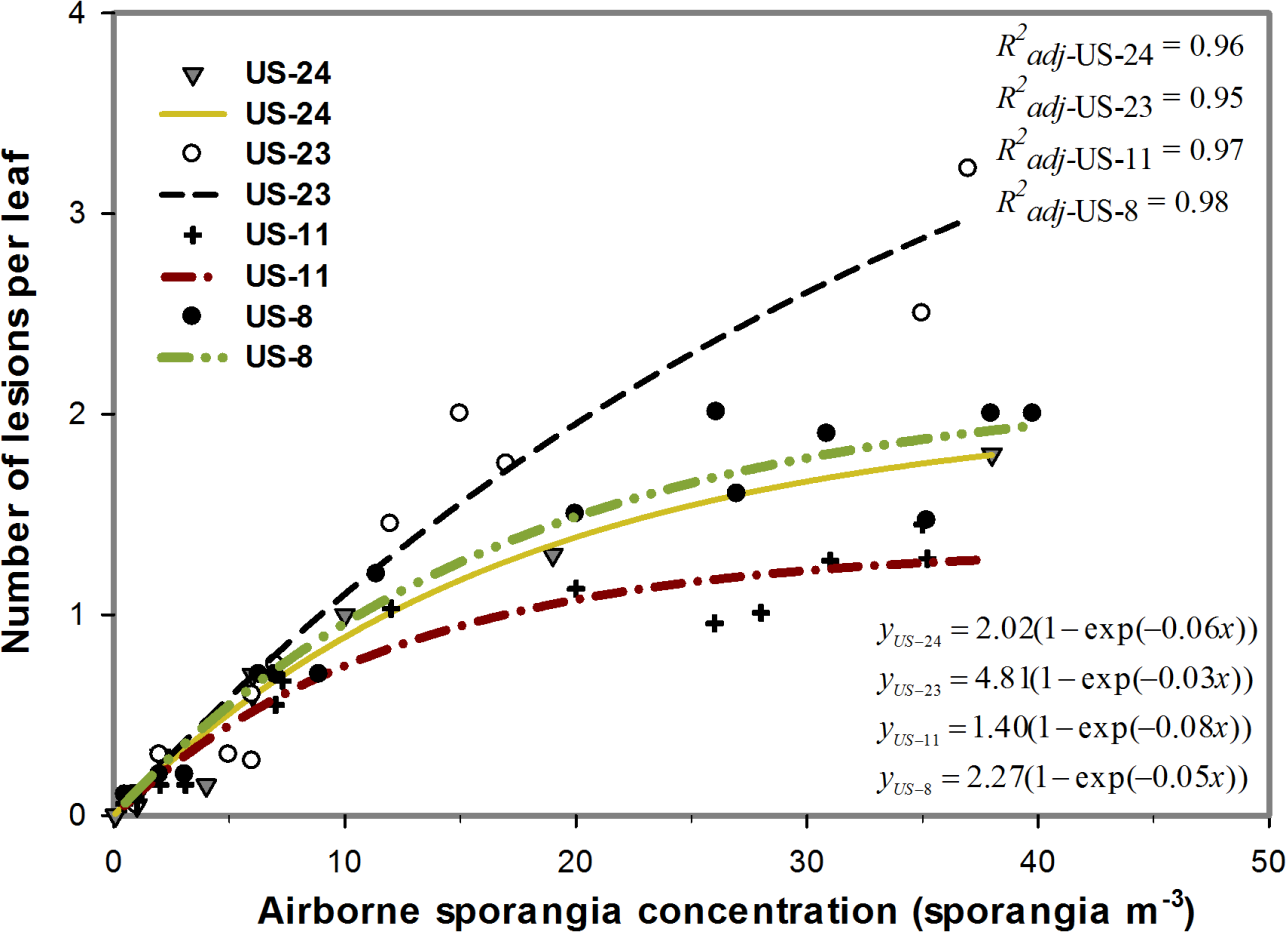
The number of lesions per leaf as a function of airborne sporangia concentration for four *Phytophthora infestans* clonal lineages. A two-parameter exponential rise to a maximum was fitted to the data. The clonal lineages tested in the study were US-8, US-11, US-23, and US-24.

The relationship between ASC and the number of lesions per leaf is shown in Fig 1. When the potato plants were exposed to a range of ASCs (between 0 and 40 sporangia m^−3^), the subsequent number of lesions per leaf increased exponentially. In this study, the maximum numbers of lesions per leaf that US-8, US-11, US-23, and US-24 could cause were 2.27, 1.40, 4.81, and 2.02, respectively. When the potato plants were exposed to a range of ASCs between 0 and 10 sporangia m^−3^ (low and medium ASCs), the number of lesions per leaf was not significantly different between clonal lineages (Tables 3 and 4, S1 Fig). However, in this study, when the ASC was above 10 sporangia m^−3^ (high ASC), the number of lesions caused by US-23 was significantly (*P* = 0.01, *P* < 0.00) higher than the number of lesions caused by the other clonal lineages (US-8, US-11, and US-24) (Table 4, S1 Fig). Also, US-8 and US-24 were not significantly different in terms of number of lesions, but US-8 caused significantly more lesions than US-11 did (Table 4, S1 Fig). An ASC of 10 sporangia m^−3^ for US-23 caused one lesion per leaf, whereas for the other clonal lineages, it took 15 to 25 sporangia m^−3^ to cause one lesion per leaf (Fig 1). In this study, clonal lineage US-23 showed higher infection efficiency than the other clonal lineages did (S1 Fig). Also, the sporangia were uniformly distributed in the growth chamber.

**Table 3:** Factorial analysis of variance of the number of lesions per leaf among clonal lineages of *Phytophthora infestans* in response to the airborne sporangia concentration in the growth chamber.

Source	Degrees of freedom	Mean square	*P-values*
**Clonal lineages**	3	0.502	0.001 **
**ASC**	2	13.195	< 2e-16 ***
**ASC:Pathotype**	6	0.420	0.00042 ***

Signif. codes: **, and *** represent *P* < 0.01, and *P* < 0.001, respectively.

**Table 4:** Results of multiple comparisons of the number of lesions per leaf among clonal lineages of *Phytophthora infestans*.

ASC	Clonal lineage		Mean difference	*P*_adj_	95% confidence	
					Lower bound	Upper bound
**Low**	US-8	US-11	−0.0033	1.00	0.6013	0.5947
		US-23	−0.0119	1.00	−0.5613	0.5375
		US-24	0.0166	1.00	0.5328	0.5661
	US-11	US-23	0.0085	1.00	−0.5697	0.5868
		US-24	−0.0200	1.00	−0.5983	0.5583
	US-23	US-24	0.0285	1.00	−0.5564	0.4993
**Medium**	US-8	US-11	0.0500	1.00	−0.8515	0.9515
		US-23	0.2000	0.99	−0.5543	0.9543
		US-24	0.3000	0.99	−0.6015	1.2015
	US-11	US-23	−0.1500	1.00	−1.0053	0.7053
		US-24	−0.2500	0.99	−1.2376	0.7376
	US-23	US-24	−0.1000	1.00	−0.9553	0.7553
**High**	US-8	US-11	0.5410	0.02*	0.0299	1.0522
		US-23	−0.4875	0.01*	−1.0505	0.0755
		US-24	0.5375	0.12	−0.0673	1.1423
	US-11	US-23	1.0285	<0.00***	0.4502	1.6068
		US-24	0.0035	1.00	−0.6154	0.6226
	US-23	US-24	−1.0250	<0.00***	−1.6875	−0.3624

Significance codes: *, and *** represent *P* < 0.05, and *P* < 0.001, respectively.

Note: The mean differences and 95% confidence levels were calculated using the Tukey HSD (honest significant difference) function. Pairwise t-test was used to calculate the adjusted P-values (P_adj_) (α = 0.05).

Airborne sporangia concentration level: ASC ≤ 5 sporangia m^-3^ is considered to be low, 5 < ASC ≤ 10 sporangia m^-3^ is considered to be medium, and ASC >10 sporangia m^-3^ is considered to be high

**Fig 2:**
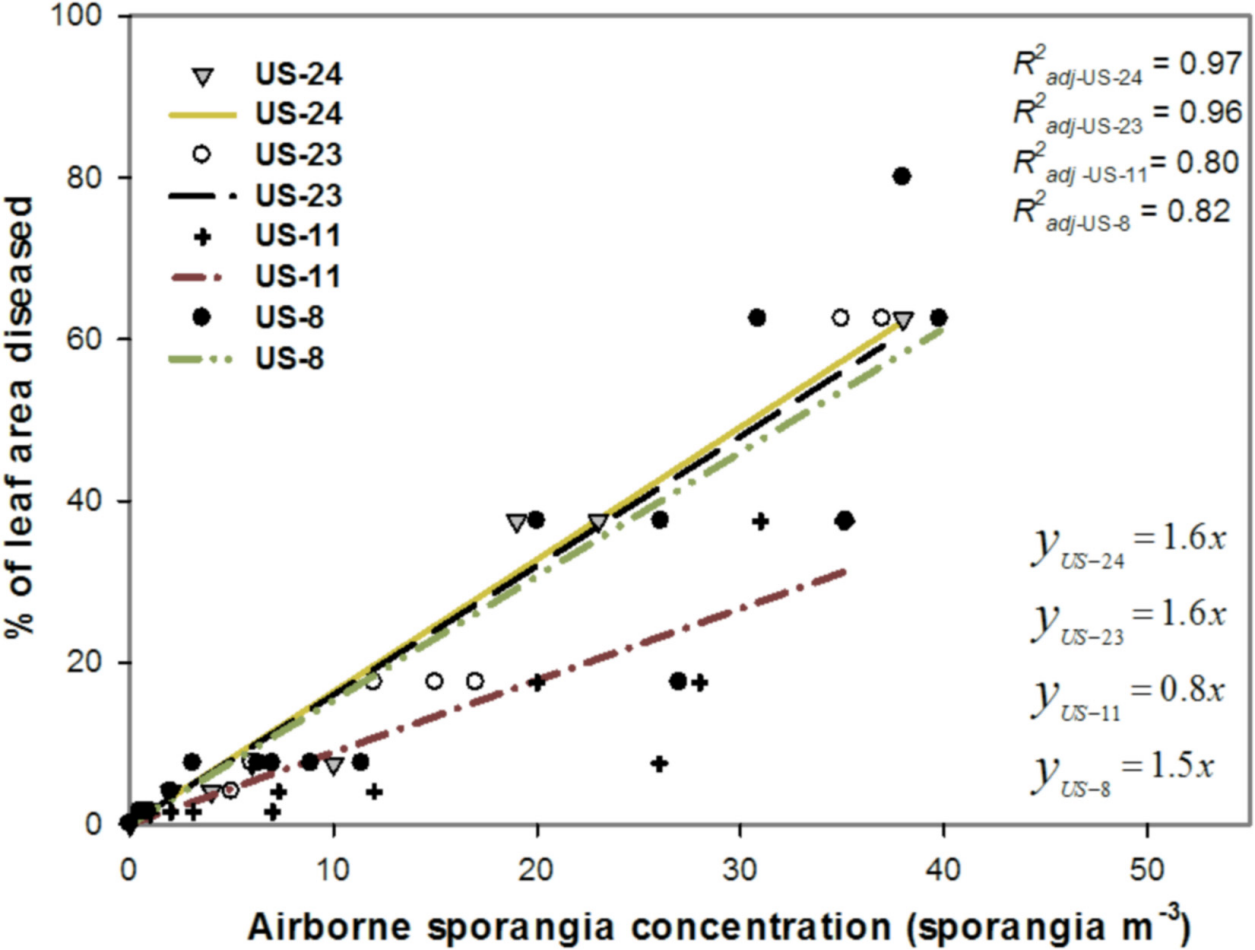
Relationship between the percentage of leaf area diseased and *Phytophthora infestans* airborne sporangia concentration. A linear function was fitted to the data. The clonal lineages tested in the study were US-8, US-11, US-23, and US-24.

The relationship between ASC and the percentage of leaf area diseased is shown in Fig 2. Late blight severity increased linearly as ASC increased. The slopes of linear regression were 1.5, 0.98, 1.6, and 1.6 for clonal lineages US-8, US-11, US-23, and US-24, respectively (Fig 2). For ASCs ranging between 10 and 40 sporangia m^−3^, the proportion of leaf area diseased caused by US-8, US-23, and US-24 increased twofold when the ASC doubled. As shown in Fig 2, an ASC of 1 sporangium m^−3^ (≈ 5 sporangia per rod) was sufficient to cause a severity of 1% for all clonal lineages. For US-8, US-23, and US-24, an ASC of 6 sporangia m^−3^ was sufficient to reach 10% leaf area diseased, whereas for US-11, 13 sporangia m^-3^ were required. Thus, US-11 showed lower aggressiveness in terms of severity than the other clonal lineages did.

### Development of the *P*. *infestans* real-time qPCR assay

#### Estimation of the number of sporangia from the number of ITS2 gene copies and specificity of the TaqMan qPCR assay

**Fig 3:**
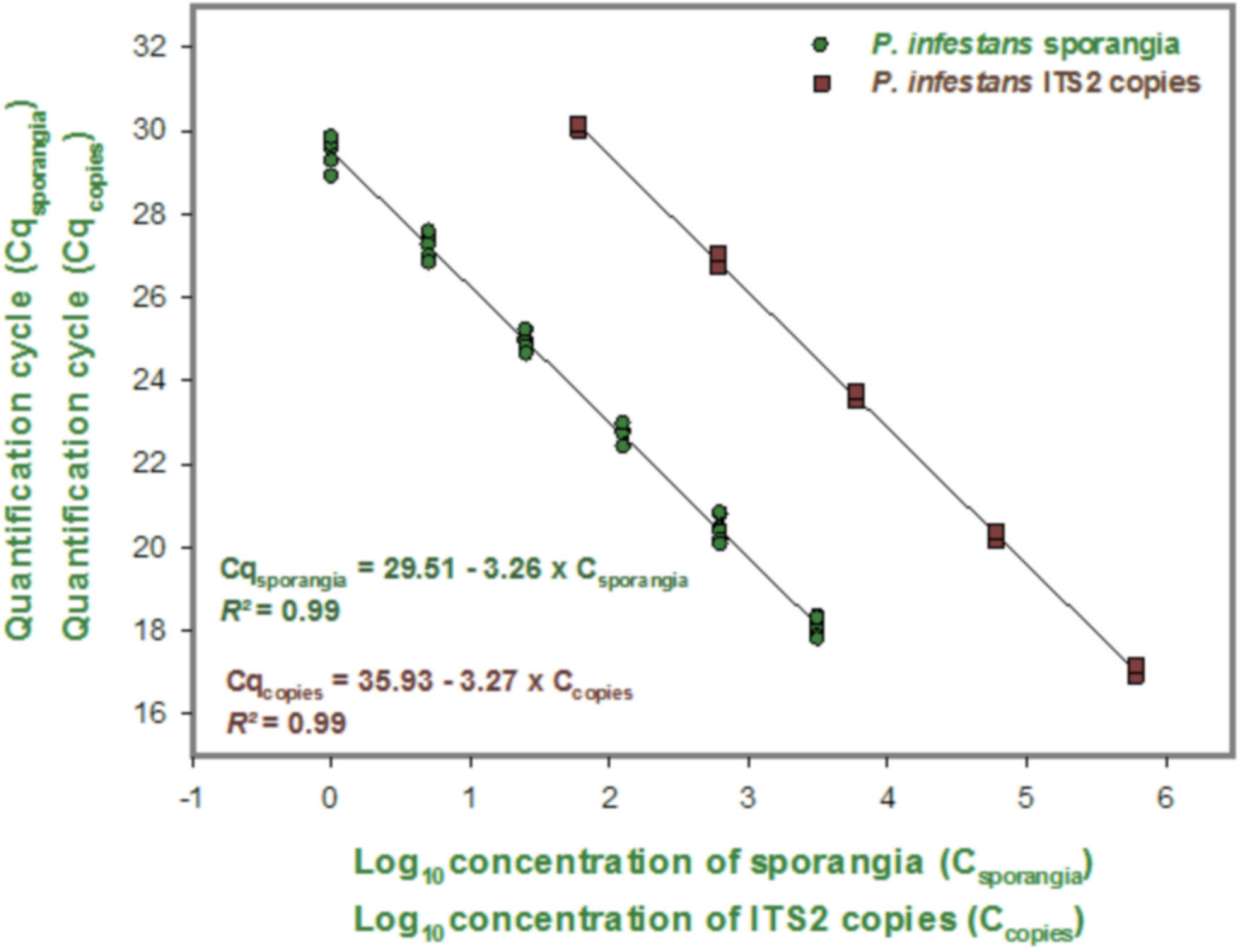
Relationship between the quantification cycle (Cq) value and the log concentration of *Phytophthora infestans* sporangia and between the Cq value and the log concentration of internal transcribed spacer 2 (ITS2) copies.

The ITS copy standard curves generated by 10-fold serial dilutions of the 1004-bp amplicon were found to be linear, with efficiency of 103.10%, a regression coefficient of 0.99, a slope of 3.27, and an intercept of 35.93. Similarly, the sporangia standard curves generated by five-fold serial dilutions of sporangia DNA extractions were also linear, with efficiency of 102.70%, a regression coefficient of 0.99, a slope of 3.26, and an intercept of 29.51 (Fig 3). A third linear regression derived from the two previous regressions was used to determine the number of ITS copies per sporangium as follows: number of ITS copies per sporangia = (1/0.011) × fraction of DNA extraction added (Fig 3). In this equation, the intercept is 0 and the slope is 0.011. The qPCR assay cross-reacted with the closely related *Phytophthora* species *P*. *mirabilis*, *P*. *ipomoeae*, *P*. *andina*, and *P*. *phaseoli* (Table 2).

### Validation of the real-time qPCR assay

**Fig 4:**
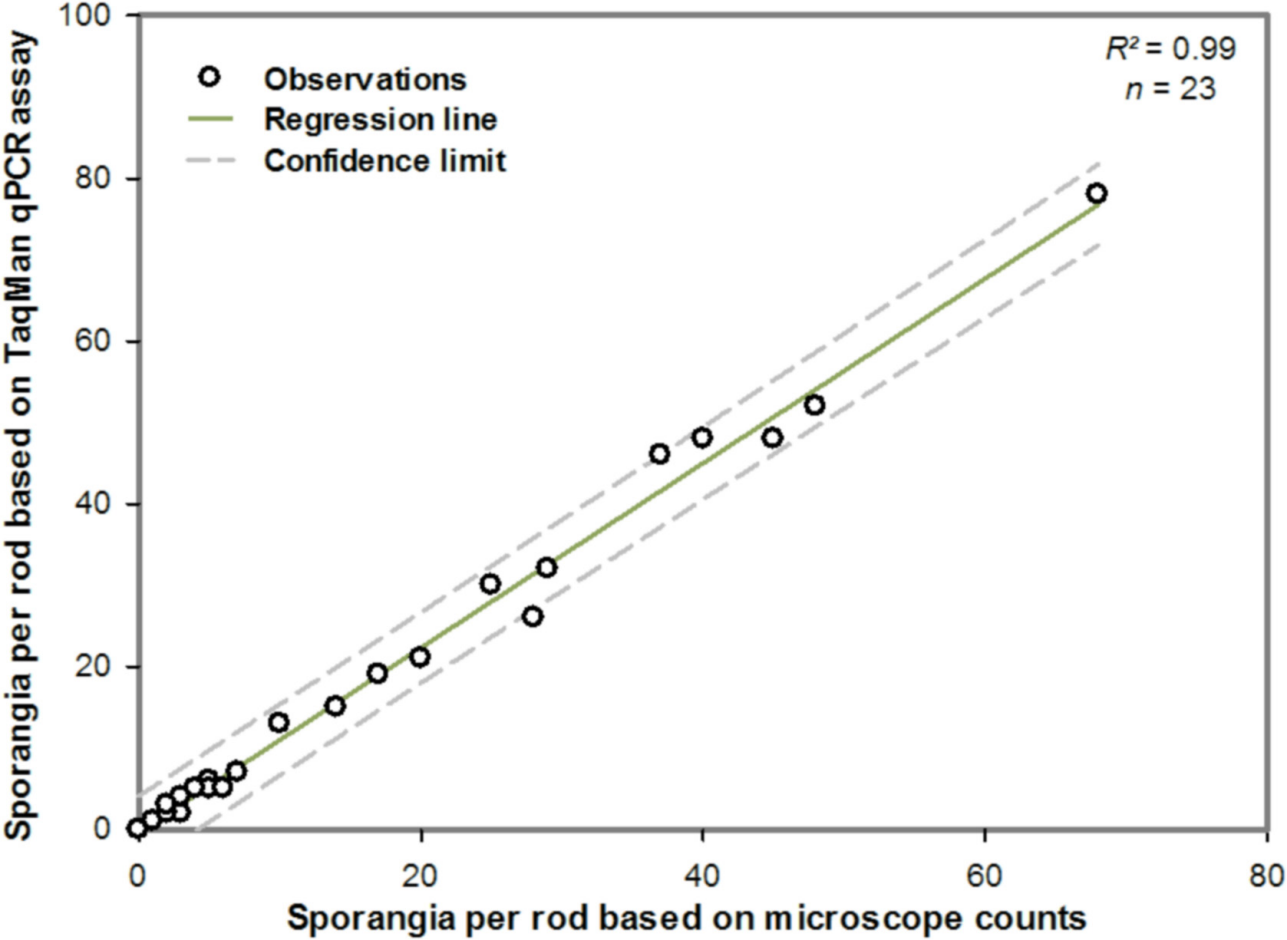
Relationship between estimates of the number of *Phytophthora infestans* sporangia deposited on silicon-greased rotating-arm sampler rods based on the *P*. *infestans* real-time qPCR assay and estimates based on microscope counts.

There is a linear relationship between the number of sporangia deposited onto the rods estimated with microscopy and the number of sporangia estimated with the TaqMan qPCR assay (*R* ^2^ = 0.99) (Fig 4). For all rods, no signals were detected from rods free of sporangia. TaqMan qPCR signals were obtained from all rods where at least one spore had been deposited.

## Discussion

The presence of new clonal lineages in different regions of North America increases concern regarding late blight control, because new clonal lineages may have unique epidemiological characteristics [10]. As mentioned in the American phytopathological society features of august 2012 [28], integrated, multifaceted strategies have the best chance for successful management of late blight. Hence, to control efficiently potato late blight in Canada and United-States, a combination of approaches in integrated manner has been used. These methods include, using certified plants and tubers, assessing pest numbers and damage, implementing better cultural practices (e.g. eliminating cull piles and volunteer potatoes), characterizing *P*. *infestans* clonal lineages, using decision support system, using spore-sampling network etc. However without information on infection efficiency, it is difficult to interpret and use airborne inoculum as late blight risk indicator. Also, the value of the information derived from spore-sampling network relies on the overall accuracy of the inoculum quantification and the determination of the infection efficiency. Information on infection efficiency will help with understanding the role of ASC in disease onset considering the epidemiological characteristics of each clonal lineage. In this study, the infection efficiency was investigated in a controlled environment, and a real-time qPCR assay for detecting and quantifying of *P*. *infestans* airborne inoculum was developed.

Under optimal infection conditions, potato late blight intensity increased as the airborne sporangia concentration (ASC) of *P*. *infestans* increased. The influence of airborne sporangia concentration was well explained by the exponential rise to a maximum model. In this study, the largest numbers of lesions that US-8, US-11, US-23, and US-24 could cause were 2.27, 1.40, 4.81, and 2.02, respectively. These results suggest that *P*. *infestans* airborne inoculum could be a good risk indicator for late blight as it was proposed in Fall et al., 2015 [8]. Also, under high airborne sporangia concentration (above 10 sporangia m^-3^) the number of lesions caused by US-23 was significantly higher than the number of lesions caused by other clonal lineages. Consequently US-23 showed higher infection efficiency than did the other clonal lineages tested in this study. Therefore, the rapid spread of US-23 and the fact that it is becoming the dominant clonal lineages in United States and Canada [10, 29, 30] may be explained in part by that lineage’s differences in terms of its capacity to produce a high number of lesions (infection efficiency). Indeed, US-8 has been the dominant clonal lineages for the past 15 years but it is gradually being replaced by US-23 [30]. Also, clonal lineages US-8 and US-24 were not significantly different in terms of infection efficiency. This result is supported by the findings of Danies et al. [10], which indicate that the pathogenicity characteristics of US-24 are similar to those of US-8. As well, the present results suggest that US-23 caused more lesions than clonal lineages US-8 and US-24 did, and US-23 was expected to cause greater diseased leaf area than US-8 and US-24 would cause.

There was a linear relationship between the diseased leaf area and the airborne sporangia concentration. The slopes of linear regression were 1.5, 0.98, 1.6 and 1.6 for clonal lineages US-8, US-11, US-23, and US-24, respectively. These results suggest that in terms of diseased leaf area there was no difference between clonal lineages US-8, US-23 and US-24. Although this may seem a contradiction with the results described above, Danies et al. [10] found that US-8 and US-24 caused larger necrotic lesions on potato plants than US-23 did. It seems that US-23 caused more lesions, but US-8 and US-24 caused larger lesions, and thus the diseased leaf area was not different between US-8, US-23, and US-24. For those three clonal lineages, an ASC of 1 and 6 sporangia m^−3^ (5 and 31 sporangia per rod) was sufficient to cause diseased leaf areas of 1% and 10%, respectively. These results can be useful for decision-making in integrated late blight management. Indeed, Stein and Kirk [4] suggested that delaying the initiation of any fungicide spray program until the estimated average foliar area with late blight reached 10% would result in disease development similar to an untreated control. Also, most fungicides and fungicide mixtures reduce late blight development as long they are applied before the diseased leaf area exceeds 1% [4]. However, the influence of temperature on whether sporangia release zoospores or instead of directly germinate may differentially influence the infection efficiency among clonal lineages [10]. Therefore, there is a need to study infection efficiency among clonal lineages in different ranges of temperature. The difference among clonal lineages could also potentially be attributed to differences in biotrophic growth that may have resulted in an underestimation of the diseased leaf area due to difficulties defining the boundaries of lesions. Overall, the findings for infection efficiency point to a clear correlation between ASC and diseased leaf areas but this still needs to be validated under field conditions. Since the action threshold suggested in this study (1 to 6 sporangia m^−3^) is low, there is need to develop a reliable, accurate and rapid ASC quantification tool other than a microscope.

Because qPCR methods can overcome the drawbacks of counting and identifying by microscopy, quantitative PCR is an appropriate tool for quantification, detection and identification of many organisms [21, 31]. To improve the detection and quantification of *P*. *infestans* airborne inoculum, a sensitive quantitative Polymerase Chain Reaction (qPCR) assay that can detect down to a single sporangium of *P*. *infestans* was developed. When *P*. *infestans* molecular assays [17, 32, 15] are evaluated for their specificity by including *P*. *phaseoli* and *P*. *mirabilis*, strong cross-reactions are often observed. The primers designed for this assay were sensitive to *P*. *mirabilis*, *P*. *phaseoli*, *P*. *ipomoeae* and *P*. *andina* since there is a high similarity among these species, sharing about 99.9% of their ribosomal DNA internal transcribed spacer regions [33]. However, *P*. *mirabilis* and *P*. *ipomoeae* do not infect potatoes or tomatoes [34–36]. The purpose of the qPCR tool is to detect and quantify airborne sporangia concentrations from potato fields or near to potato fields. In those circumstances, it can be expected that no other *Phytophthora* species or members of closely related taxa such as *Pythium* would interfere and bias the results obtained with the qPCR assay. Likewise, spores of *Pythium* would probably counted as *Phytophthora* due to their similar morphology. The analytical sensitivity evaluation showed that DNA from *P*. *infestans* spores was efficiently extracted from silicone-coated rods. In all experiments, using the qPCR assay, it was possible to detect the expected number of sporangia with a detection sensitivity of one sporangium. Thus the method of extraction is optimized and the qPCR detection is sensitive enough to detect one sporangium. The fact that no signal was observed when rods were free from *P*. *infestans* sporangia is also an indication of the reliability of the assay. Therefore, this qPCR tool allows rapid and accurate count of sporangia in contrast with microscopy. However, just as counting under the microscope, the qPCR does not distinguish between living and dead sporangia. Also, because the qPCR assay was validated only under laboratory conditions, field validation is still needed. Studies will be conducted over the next years to validate the qPCR assay with potato field samples. The spore-sampling network is already implemented at the regional scale in different potato production areas in Canada thus, the cost of network management as well as the cost of processing samplers by qPCR could be shared among growers.

This study addressed two main objectives derived from a previous study by Fall *et al*. [8], who highlighted the need to determine infection efficiency and to develop a tool for quantifying of *P*. *infestans* airborne sporangia in order to build a functional spore-sampling network to efficiently manage potato late blight. Overall, clonal lineages US-23 showed higher infection efficiency than did the other clonal lineages tested (US-8, US-11 and US-24). In addition to the traditional measurements, these results may be used to modulate the disease risk estimated by the decision support system. This study therefore provides an additional tool for late blight management and lays the groundwork for further studies to determine a field action threshold based on *P*. *infestans* ASC. To validate these findings under field conditions a qPCR assay was developed for quantifying *P*. *infestans* sporangia. Also, characteristics and management of the spore-sampling network were already described in Fall et al. 2015 [8]. Additional studies are currently underway to use airborne inoculum to determine which clonal lineage is present. Knowledge about both the amount of inoculum and the clonal lineage present can be used to adjust the fungicide scheme to the infection efficiency based on the aggressiveness of each clonal lineage. In different potato production areas in Canada, spore-sampling network has been used as the biovigilance component in order to rationalize use of fungicide, and measure effectiveness of fungicide spray programs. This network could also be used to track and map potato strains of P. infestans across Canada. For efficient control of late blight, an integrated strategy is needed and to do so, multifaceted approaches represent the best chance for more effective late blight suppression in the future [28]. This study represents an initiative towards improving interpretation of information derived from measure of airborne inoculum. The authors believe that many new technologies and approaches are needed to control late blight [28] and these include: using resistant cultivars, improving diagnostics for specific traits of *P*. *infestans*, altering planting practices, using DSSs, and measuring and characterizing airborne inoculum.

## Supporting Information

S1 Dataset Infection efficiency data for four *Phytophthora infestans* clonal lineages. (TXT)

S2 Dataset DNA-based quantification data for *Phytophthora infestans*. (XLS)

S1 Fig The mean number of lesions per leaf of four *Phytophthora infestans* clonal lineages in high, medium and level of airborne sporangia concentration (ASC). The clonal lineages tested in study were US-8, US-11, US-23, and US-24. Airborne sporangia concentration level (ASC) ≤ 5 sporangia m^-3^ is considered to be Low, 5 < ASC ≤ 10 sporangia m^-3^ is considered to be Medium, ASC >10 sporangia m^-3^ is considered to be High. The experiment was repeated 17 times for US-8, 14 times for US-11, 16 times for US-23 and 14 times for US-24.
(TIFF)

S1 Table Exogenous internal positive control (EIPC) sequence used for detection inhibition of the qPCR reaction. (PDF)
