# Supplementary figures and images for: Infection Efficiency of Four Phytophthora infestans Clonal Lineages and DNA-Based Quantification of Sporangia
Source: PLoS One. 2015 Aug 24;10(8):e0136312. doi: 10.1371/journal.pone.0136312 (PMC4547748; doi:10.1371/journal.pone.0136312)

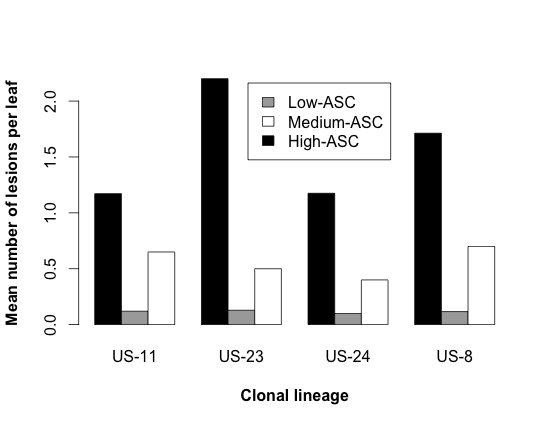

Supplement: S1 Fig — The clonal lineages tested in study were US-8, US-11, US-23, and US-24. Airborne sporangia concentration level (ASC) ≤ 5 sporangia m-3 is considered to be Low, 5 < ASC ≤ 10 sporangia m-3 is considered to be Medium, ASC >10 sporangia m-3 is considered to be High. The experiment was repeated 17 times for US-8, 14 times for US-11, 16 times for US-23 and 14 times for US-24. (TIFF) [file pone.0136312.s003.tiff]
