# Supplementary material for: Infection Efficiency of Four Phytophthora infestans Clonal Lineages and DNA-Based Quantification of Sporangia
Source: PLoS One. 2015 Aug 24;10(8):e0136312. doi: 10.1371/journal.pone.0136312 (PMC4547748; doi:10.1371/journal.pone.0136312)
Supplement: S1 Table — (PDF) [file pone.0136312.s004.pdf]

EIPC fragment sequence
TGGCACTAGAGCTGCCTGTTTCTATAGGGGGATGGGTTTTGAAAAACACTTG
ATGGGTGGGAGGACTGCATTCGAGCTGAGCGTCTCCAGAGTAAAGTACATCT
CCAACGATAAATGTAACTGGTGATCCTCTGAGTCCACCCCCTAAAGGCTAGC
TAGGACCGATCAATAGGGATAGGCAAGGTCGTCACCTCGGAACGCATAGGA
ACTGCTGACGAGAATTAAGGTCACTTTCGTAACGAAGCACTTTTCTTGCCTGT
TCGCCGCTGAGTAGCCACGTTATTATCGTCGCACCCAGATCGGGTATGAAGG
GTGTAGGACAAGTGCGGGACACAATCAAGTTAGTTGTTCGCGCCACGAGAAA
TCCGTGAAACAATACTAAGACACACAGGACTCTGAAAGCCGCTTGTATAACT
CATGTTCCGGAGGCCCGTTTCTCACGTTCGGGTTCTAGTCGCAGGGACAGAG
GAAATCACGTTCGGTTGCGGTTCTGTGCTTGG
